# Effects of a home-based low-to-moderate-intensity dance exercise program on glycemic control and quality of life in elderly patients with type 2 diabetes: a single-arm, intervention study

**DOI:** 10.1007/s13340-025-00854-6

**Published:** 2025-11-25

**Authors:** Atsushi Ujiie, Kenji Hara, Mio Kubo, Mototaka Yamauchi, Takafumi Tsuchiya, Kohzo Takebayashi, Yasuyuki Maruyama, Koshi Hashimoto

**Affiliations:** 1 Department of Diabetes, Endocrinology and Hematology, Dokkyo Medical University Saitama Medical Center, 2-1-50 Minami-Koshigaya, Koshigaya, Saitama 343-8555 Japan; 2 Department of Cardiology, Iwatsuki Minami Hospital, 2256 Kuroya, Iwatsuki, Saitama 339-0033 Japan

**Keywords:** Type 2 diabetes (T2D), Home-based dance exercise program, Health-related quality of life (HRQOL), Elderly patients

## Abstract

**Background:**

Exercise therapy improves glycemic control and reduces cardiovascular risk factors in patients with type 2 diabetes (T2D). However, access to professionally supervised programs is limited, particularly for older adults. Home-based, weather-independent, exercise options have yet to be investigated in detail.

**Objective:**

The present study examined the effects of a self-directed, low-to-moderate intensity dance exercise program performed at home on glycemic control and health-related quality of life (HRQOL) in older adults with T2D.

**Methods:**

In this single-arm, intervention study, 20 elderly patients with T2D (median age, 70.5 years) participated in a standardized, unsupervised, home-based, aerobic dance program (“DaredeMo Dance”) for at least 20 min per day for 12 weeks. The program was designed to be of low-to-moderate intensity, namely < 4 metabolic equivalents (METs). Primary outcomes were changes in HbA1c, glycoalbumin (GA), and HRQOL (assessed using SF-36v2). Secondary outcomes included body mass index (BMI), blood pressure (BP), and fasting plasma glucose (FPG).

**Results:**

After 12 weeks, significant improvements were observed in BMI (23.4 to 23.2 kg/m^2^, *P* = 0.002), systolic BP (134.0 to 125.0 mmHg, *P* = 0.004), diastolic BP (72.0 to 67.5 mmHg, *P* = 0.040), HbA1c (7.3 to 7.0%, *P* = 0.0012), and FPG (150 to 140 mg/dL, *P* = 0.034). HRQOL improved in all eight domains of SF-36v2, with significant improvements in Bodily Pain, General Health, Vitality, and Mental Health.

**Conclusions:**

A standardized, indoor, low-to-moderate intensity, dance program improved glycemic control and HRQOL in older adults with T2D. This approach offers a safe, accessible, and sustainable exercise option for those with limited access to professional guidance.

**Supplementary Information:**

The online version contains supplementary material available at 10.1007/s13340-025-00854-6.

## Introduction

Exercise therapy has been shown to improve glycemic control in patients with type 2 diabetes (T2D) [1]. Moreover, it has been reported to reduce cardiovascular risk factors, including obesity [2], visceral fat accumulation [3], insulin resistance [4, 5], dyslipidemia [6], hypertension [7], and chronic inflammation [8]. Exercise also improves quality of life (QOL) [9, 10] and may contribute to the prevention of cognitive decline [11].

Moderate-intensity aerobic exercise is recommended for patients with T2DM at least three times per week, with a total duration > 150 min, ensuring that no more than two consecutive days are free of exercise. Resistance training is also recommended two to three times per week, targeting at least five major muscle groups of the upper and lower body on non-consecutive days [12, 13]. Furthermore, balance exercises are recommended for elderly patients with T2DM to help prevent falls [14].

In practice, walking is the most commonly performed form of exercise by patients with T2D [15–17]. Other forms of physical activity, such as fitness club or community-based exercise programs, are also practiced, but walking clearly predominates.

Due to differences in age, body mass index (BMI), the presence or absence of regular exercise habits, physical capabilities, comorbidities, and diabetes-related complications among these patients, individualized exercise instructions are necessary. However, opportunities for receiving professional guidance are limited. This is partly due to a shortage of medical personnel and trainers capable of delivering exercise therapy and is further exacerbated by the national health insurance system in Japan not reimbursing patients for exercise therapy instruction.

In addition, the global COVID-19 pandemic in 2020 led to movement restrictions and stay-at-home orders in Japan, resulting in a substantial loss of exercise opportunities for many patients with T2D. Recent studies indicated the deterioration of glycemic control and weight gain [18]. Restrictions on the use of fitness clubs, club closures [19], and a decrease in daily physical activity associated with the spread of telecommuting [20] have contributed to reduced exercise levels. Furthermore, average summer temperatures in Japan have continued to increase, with the period between June and August 2024 recording the highest averages since meteorological observations began in 1898, matching records set in 2023 [21]. Such high-temperature environments increase health risks, particularly among middle-aged and elderly patients with T2D [22], and further limit opportunities for outdoor exercise. In addition to heat, other environmental factors such as heavy rainfall and natural disasters can also restrict outdoor physical activity, underscoring the need for exercise programs that can be safely implemented indoors. In the present study, a standardized instruction manual and DVD based on a dance exercise program jointly developed by dancers and medical professionals [23], modified specifically for older adults, were used. Although previous studies focused primarily on exercise therapy conducted under supervision in medical institutions or fitness clubs, this study aimed to evaluate the effectiveness of an exercise program that may be safely performed indoors independent of professional supervision and unaffected by weather conditions.

## Materials and methods

### Participants

Eligible participants were individuals aged 65 years or older at the time of consent who had HbA1c levels between 6.5 and 10.0% and had been diagnosed with T2DM. Participants with advanced diabetic complications were excluded; only those with simple diabetic retinopathy, nephropathy up to stage 3, and without severe autonomic neuropathy (defined as a coefficient of variation of the R-R interval ≥ 1%) were included. Participants were required to be capable of performing exercise of < 6 metabolic equivalents (METs; moderate intensity). The presence or absence of regular exercise habits was not considered an eligibility criterion, because 70% of participants had no prior regular exercise habits. Exclusion criteria included individuals with musculoskeletal disorders that impeded exercise, those with severe cardiovascular, hepatic, or renal diseases, individuals with ischemic heart disease, and those deemed inappropriate for participation by their attending physicians.

### Study procedures

After informed consent was obtained from the participants, their background characteristics were assessed during a screening period. Clinical laboratory evaluations included HbA1c, glycoalbumin, a complete blood count, lipid profiling, liver and renal function tests, and urinalysis. In addition, body weight (BW), body composition, chest X-ray, and electrocardiogram assessments were conducted. Participants were instructed to perform the “DaredeMo Dance” program (authored by SAM, supervised by Kenzo Yazaki, 2019, Shufunotomo Co., Ltd. Publishing, Tokyo, Japan). Participants were advised to perform the three-part program comprising a warm-up, dance exercises, and cool-down while watching the accompanying DVD for at least 20 min per day for 12 weeks. The dance program consisted of simple movements, such as dynamic upper limb motions coordinated with music, stepping in place, and side steps. The exercise intensity of the DaredeMo Dance program can be considered light to moderate. This classification is supported by a validation study of low-impact dance in elderly patients with cardiovascular disease, in which oxygen uptake during dance sessions was measured using a portable respiratory gas analyzer. The study reported values of 4.0 ± 0.2 and 3.9 ± 0.3 METs for two dance sessions, close to the anaerobic threshold (4.5 ± 0.2 METs). Heart rate responses were 105 and 97 bpm, respectively, and subjective ratings of exertion were modest, with a median Borg RPE of 11 (range 9–13). Taken together, these findings confirm that such low-impact dance corresponds to light-to-moderate aerobic exercise [23].

### Exercise monitoring

Participants recorded daily exercise time, blood pressure (BP), pulse rate (PR), BW, and their subjective physical condition using a dedicated record sheet (Supplementary information). They were instructed to avoid exercise on days they felt unwell.

### Adherence assessment

Adherence was evaluated using daily self-reported logs in which participants recorded the duration of each session (minutes). Entries of 0 min were considered “not performed.” Adherence was summarized as the number of days performed (0–84), compliance (% of 84 days), total minutes, mean minutes per performed day, and weekly totals across 12 weeks.

### Follow-up assessments

Participants underwent follow-up visits 4 and 12 weeks after starting the intervention. Clinical laboratory tests, body composition measurements using a multifrequency bioelectrical impedance analyzer (MC-780A-N, TANITA, Tokyo, Japan), and assessments of blood pressure (BP) and pulse rate (PR) were repeated. Baseline participant characteristics are shown in Table 1.

**Table 1:** Background characteristics of participants

Variable	Value
Age (years)	70.5 (67–75.5)
Sex (male/female)	13 (65%) / 7 (35%)
Duration of diabetes (years)	16.5 (9.75–22.0)
History of cardiovascular and cerebrovascular diseases (yes/no)	3 (15%) / 17 (85%)
Antihypertensive medication use (yes/no)	13 (65%) / 7 (35%)
Lipid-lowering medication use (yes/no)	12 (60%) / 8 (40%)
Smoking status (non/ex/current)	10 (50%) / 8 (40%)/ 2 (10%)
Regular exercise habit (yes/no)	6 (30%) / 14 (70%)
Diabetic retinopathy (none/simple/proliferative)	14 (70%) / 6 (30%) / 0 (0%)
Diabetic nephropathy stage (1/2/3/4/5)	14 (70%) / 6 (30%) / 0 (0%)/ 0(0%)/ 0 (0%)

A regular exercise habit was defined as ≥ 30 min/session, ≥ 2 times/week, for ≥ 1 year (based on the Ministry of Health, Labour and Welfare Japan criteria). Diabetic nephropathy stages were classified based on the criteria of the Japan Diabetes Society as follows: Stage 1 (normoalbuminuria with preserved eGFR), Stage 2 (urinary albumin excretion 30–299 mg/gCr)), Stage 3 (macroalbuminuria ≥ 300 mg/gCr), Stage 4 (eGFR < 30 mL/min/1.73 m^2^), and Stage 5 (dialysis-dependent)

### QOL assessment

Health-related quality of life (HRQOL) was assessed using the Japanese version of Short Form-36 Health Survey version 2.0 (SF-36v2), a comprehensive measure developed by Ware and colleagues [24–26]. SF-36v2 consists of 36 items that yield scores for eight domains: Physical Functioning, Role-Physical (RP), Bodily Pain (BoP), General Health (GH), Vitality (VT), Social Functioning (SF), Role-Emotional (RE), and Mental Health (MH). Based on these eight domain scores, three summary scores were also calculated: Physical Component Summary (PCS), Mental Component Summary (MCS), and Role/Social Component Summary (RCS). Each score was converted into a T-score (mean 50, standard deviation 10) according to the Japanese national standard values provided by iHope International, enabling comparisons against national norms.

### Statistical analysis

Statistical analyses were performed using the Wilcoxon signed-rank test to evaluate changes before and after the intervention. A two-sided P value < 0.05 was considered significant. Missing data were excluded from the analysis on a per-variable basis.

### Ethical considerations

The present study was conducted according to the Declaration of Helsinki and its subsequent revisions. Ethical approval was obtained from the Institutional Review Board of Dokkyo Medical University Saitama Medical Center (Dokkyo Medical University Saitama Medical Center Clinical Research Ethics Committee) on October 21, 2020 (Approval number: 2071). All participants provided written, informed consent after receiving a full explanation of the study’s purpose and procedures.

## Results

Twenty participants were enrolled in the “DaredeMo Dance” program. At baseline, median age was 70.5 [range: 67.0–75.5] years, the male/female ratio was 13/7 (Table 1), BMI was 23.4 [21.8–27.4] kg/m^2^, systolic and diastolic BPs were 131.0 [123.0–142.8] mmHg and 71.5 [67.0–78.0] mmHg, respectively, and blood HbA1c and glycoalbumin (GA) levels were 7.3% [6.9–7.6%] and 17.3% [16.4–18.3%], respectively (Table 2). Of the 20 participants, none dropped out. The median number of days performed over the 84 days was 76 (IQR 65–82), yielding a median compliance of 91.1% (IQR 77.1–97.6%); 40% completed ≥ 80 days, and 20% completed ≥ 83 days. The median total exercise time was 1804 (IQR 1,366–2,235) minutes. The median session duration on performed days was 26.8 (IQR 19.3–35.1) minutes, and the median weekly total was 150.3 (IQR 113.8–186.3) minutes. Median within-participant weekly variability (SD of weekly totals) was 38.6 (IQR 17.8–64.0) minutes. On days when exercise was performed, daily durations ranged from 4 to 75 min.

**Table 2:** Comparison of clinical parameters at 0 and 12 weeks (W)

Variable	*n*	0 W	12 W	*P* value
BMI (kg/m^2^)	19	23.4 (21.4–27.9)	23.2 (21.6–28.1)	0.002*
Systolic BP (mmHg)	18	134.0 (123.0–144.0)	125.0 (117.8–132.0)	0.004*
Diastolic BP (mmHg)	18	72.0 (67.0–78.0)	67.5 (62.0–76.3)	0.040*
PR (beats/min)	16	77.0 (67.8–83.5)	74.0 (64.0–81.0)	0.234
HbA1c (%)	19	7.3 (6.9–7.7)	7.0 (6.6–7.3)	0.012*
GA (%)	19	17.3 (16.4–18.3)	16.6 (14.7–18.4)	0.276
FPG (mg/dL)	19	150.0 (126.0–160.0)	140.0 (124.0–149.0)	0.034*
TC (mg/dL)	17	179.0 (165.0–193.0)	183.0 (157.0–200.0)	0.422
TG (mg/dL)	19	105.0 (67.0–148.0)	108.0 (78.0–146.0)	0.763
HDL-C (mg/dL)	19	59.0 (44.0–67.0)	57.0 (43.0–65.0)	0.066
LDL-C (mg/dL)	19	102.0 (90.0–125.0)	103.0 (94.0–124.0)	0.647
AST (U/L)	19	22.0 (16.0–28.0)	20.0 (17.0–25.0)	0.264
ALT (U/L)	19	16.0 (14.0–27.0)	17.0 (13.0–25.0)	0.091
ALP (U/L)	19	73.0 (57.0–90.0)	69.0 (54.0–88.0)	0.227
LDH (U/L)	19	174.0 (151.0–203.0)	165.0 (158.0–198.0)	0.116
γ-GTP (U/L)	19	24.0 (16.0–30.0)	20.0 (16.0–28.0)	0.349
BUN	19	17.0 (13.0–20.0)	16.0 (12.0–23.0)	0.479
Serum creatinine (mg/dL)	19	0.83 (0.66–1.01)	0.85 (0.68–1.01)	0.319
eGFR (mL/min/1.73 m^2^)	18	63.3 (52.6–71.2)	58.7 (52.1–69.3)	0.287
Uric acid (mg/dL)	19	5.6 (4.7–6.5)	5.8 (4.9–7.1)	0.074
Urinary albumin excretion (mg/g creatinine)	18	26.1 (12.1–105.5)	17.3 (6.1–69.9)	0.879
hs-CRP (mg/dL)	19	0.07 (0.02–0.25)	0.05 (0.03–0.11)	0.243
WBC (/µL)	19	6900 (6300–8800)	7300 (5900–8600)	0.204
RBC (10^4^/µL)	19	472.0 (435.0–507.0)	463.0 (427.0–506.0)	0.041*
Hb (g/dL)	19	14.0 (13.6–15.1)	13.9 (13.2–15.2)	0.016*
Ht (%)	19	42.5 (40.8–46.7)	43.3 (40.5–47.0)	0.087
Platelet count (10^4^/µL)	19	26.4 (21.1–29.4)	24.3 (20.0–28.4)	0.107

Data are presented as medians (interquartile range: 25th–75th percentile)

P values were calculated using the Wilcoxon signed-rank test comparing baseline (0 W) and 12 weeks (12 W). Missing data were excluded pairwise. Data are presented as medians (interquartile range: 25th–75th percentile). P values were calculated using the Wilcoxon signed-rank test comparing baseline (0 W) and 12 weeks (12 W). Asterisks (*) indicate statistically significant differences between pre- and post-intervention values (*P* < 0.05). BMI: body mass index, BP: blood pressure, PR: pulse rate, GA: glycoalbumin, FPG: fasting plasma glucose, TC: total cholesterol, TG: triglyceride, HDL-C: HDL cholesterol, LDL-C: LDL cholesterol, hs-CRP: high-sensitivity C-reactive protein, WBC: white blood cell count, RBC: red blood cell count, Hb: hemoglobin, Ht: hematocrit

Changes in physical parameters 12 weeks after the intervention are summarized in Table 2. BMI decreased significantly from 23.4 to 23.2 kg/m^2^ (*P* = 0.002).

Systolic and diastolic BPs decreased significantly from 134.0 to 125.0 mmHg (*P* = 0.004) and 72.0 to 67.5 mmHg (*P* = 0.040), respectively. Serum HbA1c levels improved significantly from 7.3 to 7.0% (*P* = 0.012), and fasting plasma glucose levels also decreased significantly from 150 to 140 mg/dL (*P* = 0.034). In contrast, GA showed no significant change after the intervention. No significant changes were observed in serum lipid profiles, including total cholesterol (C), LDL-C, HDL-C, and triglycerides. However, the red blood cell count (RBC) and hemoglobin (Hb) level showed significant decreases.

Regarding changes in QOL, improvements were observed in all eight domains of SF-36v2 (Table 3). Significant improvements were noted in BoP, GH, VT, and MH (*P* < 0.05). Of the three summary scores, both PCS and MCS improved significantly (*P* < 0.05), indicating an overall enhancement in HRQOL. In contrast, RCS showed a slight decrease (Table 4).

**Table 3:** A. Comparison of scores in eight domains of SF-36v2 at 0 and 12 weeks (W)

Variable	*n*	0 W	12 W	*P* value
PF	20	50.3 (42.1–54.4)	53.05 (49.6–56.4)	0.05
RP	20	52.35 (43.0–56.7)	56.70 (47.4–56.7)	0.08
BoP	20	48.4 (43.8–53.8)	53.8 (48.4–61.1)	0.022*
GH	20	46.7 (45.7–51.3)	53.2 (47.4–56.7)	0.002*
VT	20	52.9 (49.8–65.0)	59.0 (49.8–65.0)	0.035*
SF	20	54.9 (46.4–57.7)	57.7 (46.4–57.7)	0.474
RE	20	53.1 (37.3–56.8)	55.0 (42.8–56.8)	0.964
MH	20	52.0 (49.5–57.1)	57.1 (50.1–62.1)	0.034*

Data are presented as medians (interquartile range: 25th–75th percentile)

P values were calculated using the Wilcoxon signed-rank test comparing baseline (0 W) and 12 weeks (12 W). Missing data were excluded pairwise. Data are presented as medians (interquartile range: 25th–75th percentile). P values were calculated using the Wilcoxon signed-rank test comparing baseline (0 W) and 12 weeks (12 W). Asterisks (*) indicate statistically significant differences between pre- and post-intervention values (*P* < 0.05)

PF: Physical Functioning, RP: Role-Physical, BoP: Bodily Pain, GH: General Health, VT: Vitality, SF: Social Functioning, RE: Role-Emotional, MH: Mental Health

**Table 4:** Comparison of three summary scores of SF-36v2 at 0 and 12 weeks (W)

PCS	20	47.1 (40.1–49.9)	50.3 (48.0–53.1)	0.01*
MCS	20	52.1 (49.3–58.5)	57.2 (52.4–62.2)	0.019*
RCS	20	53.8 (45.3–58.7)	52.2 (41.4–54.6)	0.422

Data are presented as medians (interquartile range: 25th–75th percentile)

P values were calculated using the Wilcoxon signed-rank test comparing baseline (0W) and 12 weeks (12W). Missing data were excluded pairwise. Data are presented as medians (interquartile range: 25th–75th percentile). P values were calculated using the Wilcoxon signed-rank test comparing baseline (0W) and 12 weeks (12W). Asterisks (*) indicate statistically significant differences between pre- and post-intervention values (*P* < 0.05)

PCS: Physical Component Summary, MCS: Mental Component Summary, RCS: Role/Social Component Summary

## Discussion

This study demonstrated that a 12-week unsupervised, home-based, low-to-moderate-intensity, aerobic dance program (“DaredeMo Dance”) significantly improved glycemic control, BP, BMI, and HRQOL in elderly patients with T2DM. Notably,all eight SF-36v2 subscales improved, indicating positive effects on both physical and mental health domains. This study differs from previous research in several key respects. A one-way, pre-recorded video-based intervention without direct professional supervision was used, contrasting with live group instructions [27] or interactive internet-based programs [28]. Furthermore, exercise intensity was intentionally set to < 4 METs to ensure accessibility and sustainability. In contrast, most interventions reviewed by Mustapa et al. [29] incorporated combined or higher-intensity exercise. In addition, the present study focused on elderly patients with T2DM, a population underrepresented in home-based exercise research. Although Ferrer-García et al. [30] targeted elderly patients, their program was supervised and involved more complex routines. In addition, despite these modest conditions, the present program achieved significant improvements in HbA1c, BMI, BP, and HRQOL, suggesting the feasibility and effectiveness of low-barrier, standardized programs, even under environmental or resource constraints.

A distinctive feature of the present study was the use of an entertainment-oriented dance program designed for accessibility and engagement. This approach enabled safe and adherent implementation, even in the absence of trained professionals. It also supports the notion that appropriately structured interventions may succeed in regions with limited healthcare resources. Moreover, the program’s indoor format reduces exposure to environmental barriers, such as extreme weather, enhancing its safety and sustainability.

Regarding QOL outcomes, improvements were observed in all eight domains of SF-36v2, with significant gains in domains closely related to physical and emotional well-being, including BoP, GH, VT, and MH. In contrast, the SF and RE domains showed only modest changes that were not significant. One possible explanation is that baseline scores for SF and RE were already relatively high (both in the mid-50s), close to or above the Japanese national norm standardized to a mean of 50 (± 10), leaving limited room for further improvement (ceiling effect). In addition, because the DaredeMo Dance program was performed individually at home using a DVD without supervision, it may have provided psychological enjoyment and a sense of accomplishment, but it did not directly stimulate interpersonal interactions or social roles, which are more closely reflected in SF and RE. Moreover, the intervention lasted only 12 weeks, which may have been insufficient to elicit measurable changes in psychosocial domains, since such aspects typically require longer-term interventions to improve. In the present study, the RCS score decreased slightly following the intervention, which may be partly attributed to the absence of significant improvements in its component subscales (RP, SF, and RE). Notably, “lack of time” is often cited as a major barrier to regular exercise by patients with T2D [31]. With respect to clinical significance, the observed improvements of + 3.3 points in PCS and + 5.1 points in MCS exceed commonly referenced MCID thresholds in the literature (approximately 2–5 points) [32, 33]. At the domain level, improvements in BoP, GH, VT, and MH also fell within or above this MCID range, supporting their interpretation as clinically meaningful. In contrast, changes in RE did not reach the domain-specific MCID threshold, indicating limited clinical significance in this areas.

Although the intensity of the program was low, participants achieved significant improvements in HbA1c, fasting plasma glucose, BMI, and both systolic and diastolic BPs. While HbA1c levels showed significant improvement, GA did not change significantly. This discrepancy may reflect intrinsic differences between the biomarkers: HbA1c reflects longer-term glycemic exposure (approximately 8–12 weeks), whereas GA is more sensitive to short-term glucose fluctuations and influenced by albumin kinetics and metabolism. In individuals without overt obesity, the modulatory effect of adiposity on GA may be attenuated, reducing the impact of BMI as a confounder. Nonetheless, the relatively short intervention duration and small sample size may have also constrained the ability to detect meaningful changes in GA. This aligns with previous findings by Umpierre et al. [1] and the American Diabetes Association position statement by Colberg et al. [34], which emphasize frequency and sustainability over intensity. Similarly, Yates et al. [35] reported that low-intensity walking integrated into daily routines was effective for diabetes prevention. The present results support these conclusions and highlight the role of habit formation in exercise adherence.

The dance component may have contributed to psychological enjoyment and a sense of accomplishment, reducing resistance to exercise and enhancing motivation. The program’s association with a public figure may also have fostered trust and engagement.

In addition, the significant decreases observed in RBC and Hb may reflect exercise-induced plasma volume expansion, leading to hemodilution. The DaredeMo Dance program has been classified as light-to-moderate aerobic exercise [23], and thus can be regarded as a form of endurance-type activity. As noted by Convertino et al. [36], endurance training increases blood volume, particularly plasma volume, which may dilute circulating blood components. Such physiological adaptations may, therefore, also have occurred in the present study.

There are several limitations that need to be acknowledged. This study involved a small sample size, was conducted at a single center, and used a single-arm design over a short duration, thereby limiting generalizability. Because of the single-arm design, potential placebo effects or natural improvements over time cannot be ruled out. Participants were relatively health-conscious and recruited from a university hospital, which may have introduced a selection bias. The participant group was also relatively elderly and mildly obese; therefore, the applicability of these results to patients with higher BMIs or those receiving insulin therapy remains uncertain. In addition, exercise adherence was assessed through self-reported logs, and no objective measures of physical activity or exercise intensity were collected. Future studies should address these limitations by conducting multicenter, randomized, controlled trials with more diverse populations and by incorporating objective metrics, such as accelerometers or continuous glucose monitors, to confirm the present findings. Despite these limitations, the major contribution of this study lies in demonstrating that a standardized, home-based, exercise program, implemented without professional supervision and resilient to environmental conditions, supported improvements in glycemic control and QOL in older patients with T2DM. Similar programs may be valuable for promoting sustainable exercise interventions in community and home-based care systems.

## Supplementary Information

Below is the link to the electronic supplementary material.

Supplementary Material 1: The Workout Log Sheet presents the dedicated self-recording sheet used by participants to document their daily physical condition (selected from predefined options), blood pressure, pulse rate, body weight, and the duration of “DaredeMo Dance” performed (recorded in minutes).
