# Supplementary figures and images for: Effects of a home-based low-to-moderate-intensity dance exercise program on glycemic control and quality of life in elderly patients with type 2 diabetes: a single-arm, intervention study
Source: Diabetol Int. 2025 Nov 25;17(1):6. doi: 10.1007/s13340-025-00854-6 (PMC12647483; doi:10.1007/s13340-025-00854-6)

## Slide 1
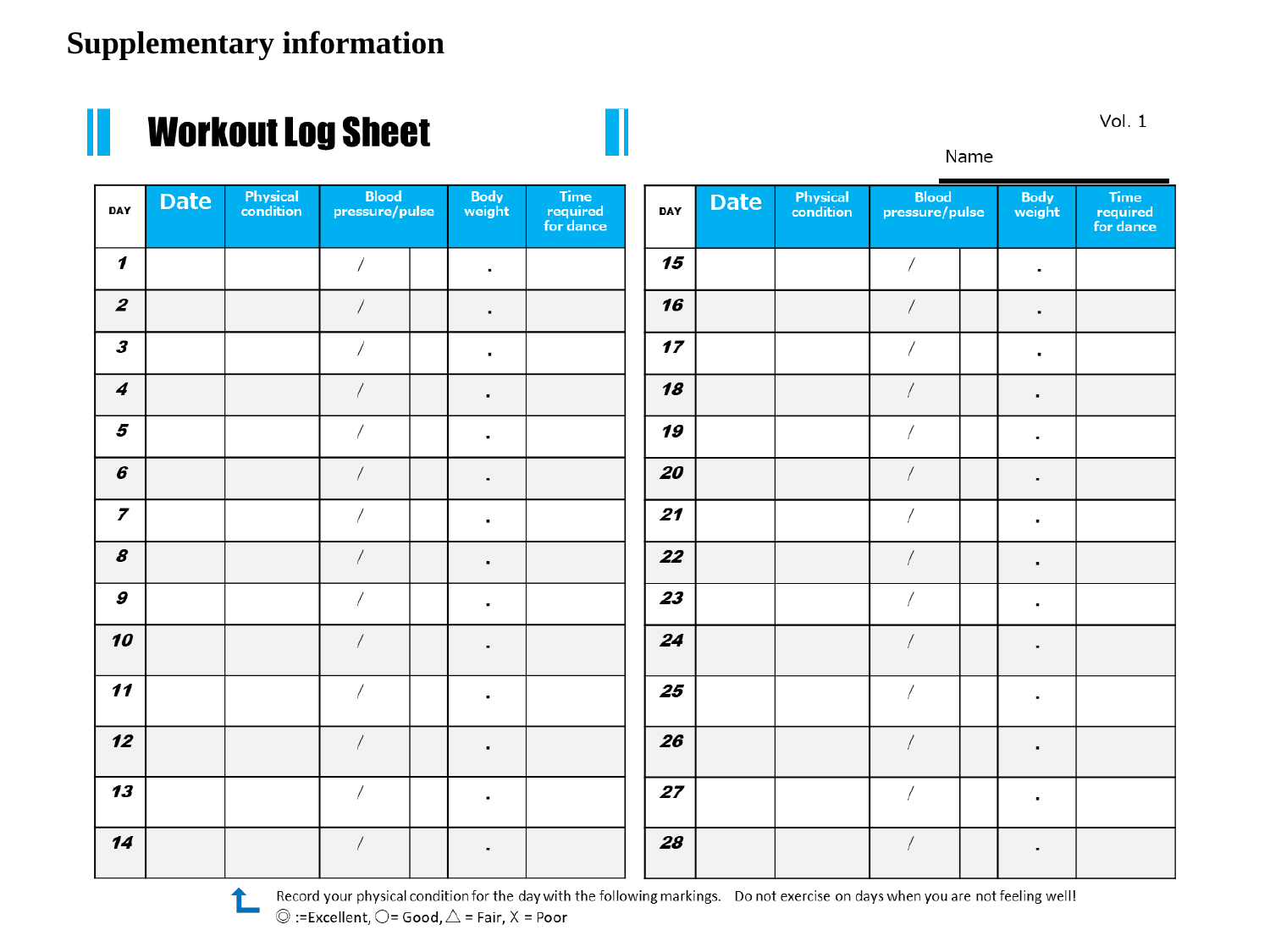

Supplementary information

Supplement: Supplementary file 1 — Supplementary Material 1: The Workout Log Sheet presents the dedicated self-recording sheet used by participants to document their daily physical condition (selected from predefined options), blood pressure, pulse rate, body weight, and the duration of “DaredeMo Dance” performed (recorded in minutes). [file 13340_2025_854_MOESM1_ESM.pptx]
